# Comparative analysis of robotic, laparoscopic, and open ileal pouch–anal anastomosis outcomes: retrospective cohort study

**DOI:** 10.1093/bjsopen/zraf084

**Published:** 2025-07-31

**Authors:** Tommaso Violante, Sacha P Broccard, Marco Novelli, Luca Stocchi, Dorin T Colibaseanu, Michelle F DeLeon, Kevin T Behm, Nitin Mishra, David W Larson, Amit Merchea

**Affiliations:** Division of Colon and Rectal Surgery, Mayo Clinic, Rochester, Minnesota, USA; School of General Surgery, Bologna University, Bologna, Italy; Division of Colon and Rectal Surgery, Mayo Clinic, Jacksonville, Florida, USA; ORSI Academy, Gent, Belgium; Division of Colon and Rectal Surgery, Mayo Clinic, Jacksonville, Florida, USA; Division of Colon and Rectal Surgery, Mayo Clinic, Rochester, Minnesota, USA; Department of Statistics, University of Bologna, Bologna, Italy; Division of Colon and Rectal Surgery, Mayo Clinic, Jacksonville, Florida, USA; Division of Colon and Rectal Surgery, Mayo Clinic, Jacksonville, Florida, USA; Division of Colon and Rectal Surgery, Mayo Clinic, Jacksonville, Florida, USA; Division of Colon and Rectal Surgery, Mayo Clinic, Rochester, Minnesota, USA; Division of Colon and Rectal Surgery, Mayo Clinic, Phoenix, Arizona, USA; Division of Colon and Rectal Surgery, Mayo Clinic, Rochester, Minnesota, USA; Division of Colon and Rectal Surgery, Mayo Clinic, Jacksonville, Florida, USA

**Keywords:** robotic, laparoscopy, IPAA, ulcerative colitis, IBD, pouch

## Abstract

**Introduction:**

Ileal pouch–anal anastomosis (IPAA) is a common surgical procedure for patients with ulcerative colitis or familial adenomatous polyposis. This study compared the outcomes of robotic, laparoscopic, and open IPAA techniques, with a focus on surgical complications and pouch failure rates.

**Methods:**

A retrospective study was conducted of patients who underwent IPAA at three Mayo Clinic locations between 2015 and 2020. Data on patient demographics, surgical details, and postoperative outcomes were collected and compared across the three surgical approaches. Pouch failure was defined as the need for pouch excision or a diverting loop ileostomy.

**Results:**

In all, 401 patients underwent IPAA with either an open (149, 37.2%), robotic (145, 36.2%), or laparoscopic (107, 26.7%) technique. The overall rate of pouch failure was 6.5% and did not differ significantly between the three surgical approaches. Compared with laparoscopy, robotic IPAA was associated with a lower conversion rate to open surgery (1.4 *versus* 17.8%; *P* < 0.0001) and fewer 30-day readmissions (15.9% *versus* 28.0%; *P* = 0.02). However, robotic and laparoscopic IPAA approaches had higher rates of venous thromboembolism/pulmonary embolism and readmission than the open approach. Pouchitis was the most common cause of pouch failure across all surgical techniques.

**Conclusion:**

Robotic IPAA had lower conversion and reduced 30-day admission rates compared with a laparoscopic approach. However, open surgery had lower rates of 30-day readmission and rates thromboembolism than robotic IPAA. The surgical approach itself does not appear to significantly impact long-term pouch failure rates.

## Teaser

This study compared robotic, laparoscopic, and open ileal pouch–anal anastomosis (IPAA). Although overall pouch failure rates were similar across the three approaches, robotic IPAA had lower conversion rates to open surgery and fewer 30-day readmissions than laparoscopic IPAA. Open surgery was associated with a lower incidence of thromboembolic events. The surgical approach did not significantly affect long-term pouch failure.

## Introduction

First introduced in 1978^1^, restorative proctocolectomy with ileal pouch–anal anastomosis (IPAA) has rapidly become the standard for patients with ulcerative colitis (UC) and familial adenomatous polyposis (FAP)^2^.

Over the past two decades, various innovations have enhanced this procedure. The adoption of laparoscopy reduced short-term complications, length of hospital stay, and blood loss while improving cosmesis and hastening the return of bowel function^3–6^. More recently, robotic surgery has been embraced for pelvic procedures due to its advantages in navigating the narrow pelvis, improved surgeon ergonomics, enhanced instrument dexterity, and superior three-dimensional visualization^7^.

Despite these technological advances, IPAA remains associated with a risk of pouch failure, defined as loss or compromised function of the ileal reservoir requiring either pouch excision/revision or construction of a new diverting ostomy^8,9^. In the current era of robotic surgery, there remains a lack of comprehensive comparison between the three available surgical modalities (open, laparoscopic, and robotic), with even less evidence regarding their associated pouch failure rates^10^.

The aim of this study was to determine the short-term differences between surgical approaches for the proctectomy stage of IPAA from a single healthcare system with standardized perioperative protocols. In addition, the pouch failure rates across these three techniques were defined and analysed to determine whether there any differences across the surgical approaches and to identify factors associated with pouch failure.

## Methods

This retrospective study was conducted in accordance with the STROBE guidelines. After obtaining Institutional Review Board approval (Mayo Clinic IRB 23-000779-07), patient data were collected between January 2015 and January 2020 at three Mayo Clinic locations: Mayo Clinic Rochester, Mayo Clinic Florida, and Mayo Clinic Arizona. The study included patients who underwent either a proctocolectomy with IPAA and diverting loop ileostomy (DLI; as part of a two-stage approach) or a proctectomy with IPAA and DLI (as part of a three-stage approach). The surgical approaches used were robotic, laparoscopic, or open. Patients who underwent a two-stage hand-assisted approach were included in the open category because the proctectomy portion of the operation was undertaken as an open technique via the hand port incision.

Pouch failure, requiring pouch excision or DLI, was the primary outcome. Secondary outcomes focused on comparing short-term postoperative results among the different surgical approaches.

Various patient characteristics were recorded, including sex, age at IPAA construction, body mass index (BMI), underlying disease, smoking status, diabetes, and the preoperative use of steroids, immunomodulators, and biologics within 30 days of surgery. Surgical data were also collected, including operative time, estimated blood loss (EBL), and intraoperative complications.

Postoperative outcomes were tracked and included: length of hospital stay; detailed 30-day morbidity (ileus, defined as longer than 3 days with nil per os or requiring nasogastric tube insertion; small bowel obstruction requiring reoperation; venous thromboembolism (VTE) and pulmonary embolism (PE); superficial surgical site infection; urinary tract infection; anastomotic leak, defined as the radiological presence of a leak; and pelvic collection, when no leak was evident); 30-day readmission and reoperation rates; 30-day mortality; DLI reversal rate; and time to DLI reversal. The minimally invasive IPAA techniques used in the study have been described in detail previously^11,12^. All robotic procedures were performed using the robotic Da Vinci Xi Surgical System (Intuitive Surgical, Sunnyvale, CA, USA).

The interval between IPAA creation and ileostomy closure was at the discretion of the operating surgeon but was typically scheduled approximately 3 months after IPAA.

The choice of surgical approach was left to the discretion of the operating surgeon. All operations were completed by 16 board-certified colorectal surgeons who had extensive experience in IPAA surgery.

### Statistical analysis

Statistical analysis involved descriptive and inferential methods. Categorical data are presented as counts (percentages) and continuous data are presented as the median with interquartile range (i.q.r.). Categorical variables were compared between groups using χ^2^ or Fisher's exact tests, whereas the significance of differences in continuous variables was assessed using the Kruskal–Wallis test. Post hoc analyses (χ^2^, Fisher's exact, or Mann–Whitney *U* tests) identified specific group differences.

**Fig. 1:**
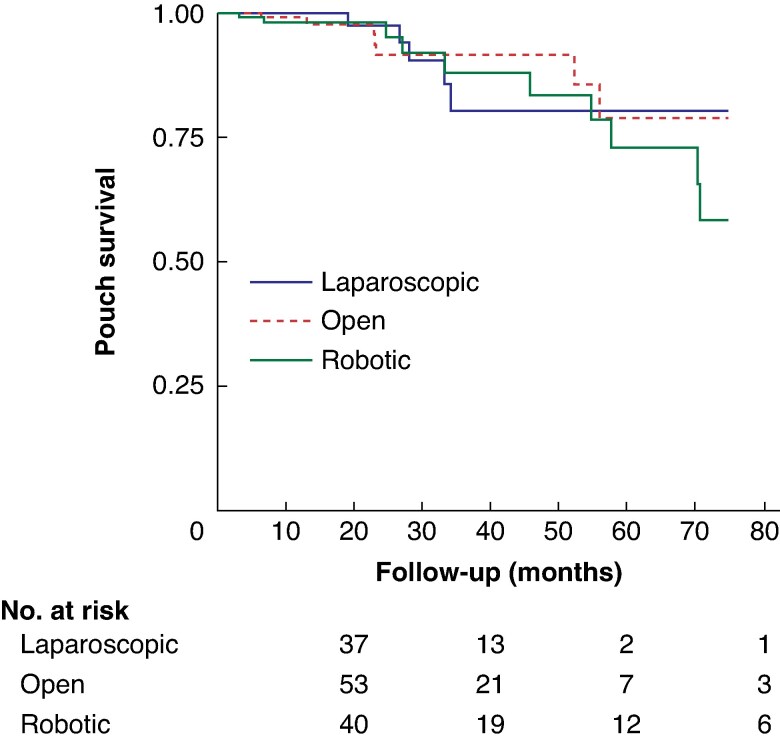
Survival analysis of pouch failure for laparoscopic, open, and robotic surgery

In the initial phase of the study, an approach-stratified analysis of Kaplan–Meier survival curves over a 6-year span was conducted (*Fig. 1*). In addition, a Cox regression model was used to identify factors associated with pouch failure. The control variables considered included age, BMI, sex, the use of preoperative steroids and immunomodulators within 30 days of surgery, underlying disease, American Society of Anesthesiologists (ASA) grade, and number of IPAA stages. To mitigate the impact of potential model misspecifications, robust standard errors were used^13^. The proportional hazards assumption was assessed using both graphical and inferential methods; no violation was detected^14^.

For operative and postoperative outcomes, multivariable analyses were performed using linear regression for continuous variables (for example, length of hospital stay, operative time, and EBL) and logistic regression for binary outcomes (for example, 30-day readmission). The models incorporated the same control variables as those used in the Cox model. For the variable conversion to open surgery, only patients undergoing a robotic or laparoscopic approach were included. Robust standard errors were used in all analyses.

## Results

### Patient characteristics

This study included 401 patients, with most undergoing open (149, 37.2%) or robotic (145, 36.2%) IPAA; 107 patients (26.7%) underwent laparoscopic IPAA. No differences were found in sex, age, or ASA grade (*Table 1*). However, the open and robotic groups had a significantly higher BMI than the laparoscopic group (*P* = 0.02). Most patients underwent IPAA because of ulcerative colitis (92.0%), with only a minority (8.0%) needing the same procedure for polyposis.

**Table 1:** Patient characteristics for the laparoscopic, robotic, and open IPAA groups

	Robotic (*n* = 145)	Laparoscopic (*n* = 107)	Open (*n* = 149)	*P*
**Sex**				
Male	86 (59.3%)	54 (50.5%)	90 (60.4%)	0.2*
Female	59 (40.7%)	53 (49.5%)	59 (39.6%)	
Age (years), median (i.q.r.)	37.2 (28.0–49.6)	37.7 (27.1–51.1)	37.5 (28.5–50.3)	1.0†
BMI (kg/m^2^), median (i.q.r.)	24.9 (21.7–28.0)	24.6 (21.0–27.7)	26 (23–29)	0.02†
**ASA grade**				0.6‡
I	3 (2.1%)	3 (2.8%)	22 (14.8%)	
II	115 (79.3%)	85 (79.4%)	96 (64.4%)	
III	27 (18.6%)	19 (17.8%)	31 (20.8%)	
**Underlying disease**				0.9*
FAP	10 (6.9%)	11 (10.3%)	11 (7.4%)	
Ulcerative colitis	135 (93.1%)	96 (89.7%)	138 (92.6%)	
Active smoker at surgery	6 (4.1%)	0 (0%)	6 (4.0%)	0.07‡
Diabetes	5 (3.5%)	0 (0%)	6 (4.1%)	0.08‡
Preoperative steroids within 30 days of surgery	10 (6.9%)	11 (10.3%)	11 (7.4%)	0.6*
Preoperative immunomodulators within 30 days of surgery	18 (12.4%)	3 (2.8%)	9 (6.0%)	0.01‡
Preoperative biologics within 30 days of surgery	22 (15.2%)	3 (2.8%)	15 (10.1%)	0.005‡

Values are *n* (%) unless otherwise stated. IPAA, ileal pouch–anal anastomosis; min, minutes; i.q.r., interquartile range; BMI, body mass index; ASA, American Society of Anesthesiologists; FAP, familial adenomatous polyposis. *χ^2^ test; †Kruskal–Wallis test; ‡Fischer's exact test.

Although no significant differences were observed in most co-morbidities, patients who underwent robotic IPAA were significantly more often using immunomodulators within 30 days of the procedure compared with patients in the laparoscopic and open groups (12.4 *versus* 2.8 and 6.0%, respectively; *P* = 0.01). In addition, the use of preoperative biologics was significantly higher in the robotic group than in the laparoscopic and open groups (15.2 *versus* 2.8 and 10.1%, respectively; *P* = 0.005).

### Operative variables

Operative data (*Table 2*) revealed that most patients underwent a three-staged approach (69.3%), with the robotic group having a statistically significant higher rate of patients undergoing a two-staged procedure than the laparoscopic and open surgery groups (38.6 *versus* 29.0 and 24.2%, respectively; *P* = 0.02). Twenty-seven per cent of patients underwent hand-assisted procedures and were included in the open patient group given that the proctectomy was performed in an open fashion through the hand port incision. Open IPAA had the shortest operative time (213 (i.q.r. 133) minutes (min)), followed by laparoscopic (257 (i.q.r. 159) min), and robotic (300 (i.q.r. 100) min) IPAA (*P* = 0.0001). EBL was significantly lower for the robotic group (75 (i.q.r. 50) ml) than for the open (100 (i.q.r. 75) ml) and laparoscopic (75 (i.q.r. 125) ml) groups (*P* = 0.0001). Conversion to open surgery was significantly lower in the robotic than laparoscopic group (1.4 *versus* 17.8%; *P* < 0.0001).

**Table 2:** Operative and postoperative outcomes of laparoscopic, robotic, and open IPAA techniques

	Robotic (*n* = 145)	Laparoscopic (*n* = 107)	Open (*n* = 149)	*P*
Two-staged approach	56 (38.6%)	31 (29.0%)	36 (24.2%)	0.020*
Three-staged approach	89 (61.4%)	76 (71.0%)	113 (75.8%)	0.021*
Operative time (min), median (i.q.r.)	300 (243–343)	257 (189–348)	213 (157–290)	<0.001†
EBL (ml), median (i.q.r.)	75 (50–100)	75 (50–175)	100 (75–150)	<0.001†
Conversion to open surgery	2 (1.4%)	19 (17.8%)	–	<0.001*
Length of hospital stay (days), median (i.q.r.)	4 (3–6)	3 (2–4)	4 (4–6)	<0.001†
30-day complications	47 (32.4%)	41 (38.3%)	38 (25.5%)	0.091‡
Ileus	22 (15.2%)	27 (25.3%)	24 (16.1%)	0.100‡
Small bowel obstruction	8 (5.5%)	6 (5.6%)	4 (2.7%)	0.411*
VTE/pulmonary embolism	8 (5.5%)	8 (7.5%)	1 (0.7%)	<0.001*
Superficial SSI	2 (1.4%)	2 (1.9%)	4 (2.7%)	0.901*
Urinary tract infection	4 (2.8%)	5 (4.7%)	2 (1.3%)	0.237*
Anastomotic leak	15 (10.3%)	10 (9.4%)	10 (6.7%)	0.539‡
Pelvic collection	9 (6.2%)	7 (6.5%)	7 (4.7%)	0.810‡
30-day readmission	23 (15.9%)	30 (28.0%)	10 (6.7%)	<0.001‡
30-day reoperation	6 (4.1%)	7 (6.5%)	2 (1.3%)	0.084*
30-day death	0 (0%)	0 (0%)	1 (0.7%)	1.000*
Days with DLI, median (i.q.r.)	97 (86–109)	95 (83–107)	100 (76–124)	0.207
DLI reversal	136 (94.4%)	103 (97.2%)	146 (98.7%)	0.138*
Follow-up from DLI closure (months), median (i.q.r.) (months)	9.1 (3.7–23.3)	14.1 (4.4–27.5)	12.3 (5.9–28.6)	0.005†
**Pouch failure**				
Total	12 (8.4%)	5 (4.7%)	9 (6.1%)	0.712‡
Early (within 6 months)	3 (33.3%)	2 (40.0%)	5 (55.6%)	0.549*
Late (after 6 months)	8 (66.7%)	3 (60.0%)	4 (44.4%)	

Values are *n* (%) unless otherwise stated. IPAA, ileal pouch–anal anastomosis; min, minutes; i.q.r., interquartile range; EBL, estimated blood loss; VTE, venous thromboembolism; SSI, surgical site infection; DLI, diverting loop ileostomy. *Fischer's exact test; †Kruskal–Wallis test; ‡χ^2^ test.

### Postoperative outcomes

The laparoscopic group had a significantly shorter median length of hospital stay (3 days) than the robotic and open groups (4 days each); this advantage was offset by a significantly higher 30-day readmission rate (28%) in the laparoscopic group than in the robotic and open groups (15.9 and 6.7%, respectively; *P* < 0.0001; Table 2). The higher readmission rate in the laparoscopic group was primarily driven by an increased incidence of small bowel obstruction/ileus and intra-abdominal/pelvic abscess. The most common causes for 30-day readmission across all surgical approaches were small bowel obstruction and ileus (22 patients), pelvic abscess (16), high ostomy output with dehydration (14), superficial surgical site infection (3), VTE (3), pain (2), anastomotic leak (1), bleeding (1), and psychosis (1). Of the 22 patients presenting with small bowel obstruction and ileus, 18 were in the minimally invasive surgery groups (laparoscopic and robotic), with obstruction predominantly at the stoma site. The robotic and laparoscopic groups had a higher rate of VTE/PE than the open group (*P* < 0.0001). However, there were no significant differences in most 30-day complications, including rates of ileus, small bowel obstruction, superficial surgical site infection, urinary tract infection, anastomotic leak, pelvic collection, 30-day reoperation, and 30-day mortality. An IPAA could not be successfully created in 3 patients (0.7%). In addition, no differences were observed in the number of days with a DLI or DLI reversal rate. A statistically significant difference emerged in the length of follow-up after DLI closure, with the laparoscopy and open groups having the longest follow-up (median 14.1 and 12.1 months, respectively, *versus* 9.1 months in the robotic group; *P* = 0.005).

The overall pouch failure rate (6.5%) showed no significant variation between surgical techniques. Pouchitis was the most frequent cause of failure (7 patients), followed by IPAA fistula (6), incontinence (3), Crohn's disease of the pouch (3), IPAA leak (3), pouch stricture (2), and recurrent volvulus (1).

### Post hoc analysis

Post hoc analysis revealed several key distinctions between the three surgical approaches (*Table 3*). The open and robotic groups had a higher BMI than the laparoscopic group (*P* = 0.008 and *P* = 0.01, respectively). Preoperative immunomodulator use was more frequent in among patients in the robotic than laparoscopic IPAA group (*P* = 0.006). Similarly, biologics were more commonly used by patients undergoing open and robotic surgery than those undergoing a laparoscopic approach (*P* = 0.03 and *P* = 0.001, respectively). Two-staged IPAA was more prevalent in the robotic group, whereas the rate of a three-staged approach was higher in the open group (*P* = 0.008). Operative time and EBL varied significantly across all three approaches. Laparoscopic IPAA resulted in a significantly shorter length of hospital stay than both open and robotic IPAA (*P* < 0.0001 and *P* = 0.02, respectively). The robotic and laparoscopic groups experienced more VTE events than the open group (*P* = 0.02 and *P* = 0.005, respectively). The 30-day readmission rate was notably higher in the laparoscopic group than in the robotic and open groups (*P* = 0.02 and *P* < 0.0001, respectively). The robotic group had a shorter follow-up duration than the open group (*P* = 0.03).

**Table 3:** Post hoc analysis of laparoscopic, robotic, and open IPAA techniques

	Laparoscopic	Robotic	Open	*P*		
				Robotic *versus* laparoscopic	Robotic *versus* open	Open *versus* laparoscopic
BMI (kg/m^2^), median (i.q.r.)	24.6 (21.0–27.7)	24.9 (21.7–28.0)	26 (23–29)	0.014*	0.093*	0.008*
Preoperative immunomodulators within 30 days of surgery	3 (2.8%)	18 (12.4%)	9 (6.0%)	0.006†	0.064‡	0.218‡
Preoperative biologics within 30 days of surgery	3 (2.8%)	10 (6.9%)	9 (6.0%)	0.001†	0.2†	0.034†
Two-staged approach	31 (28.9%)	56 (38.6%)	36 (24.2%)	0.115‡	0.008*	0.212‡
Three-staged approach	76 (71.1%)	89 (61.4%)	113 (75.8%)	0.138‡	0.008*	0.221‡
Operative time (min), median (i.q.r.)	257 (189–348)	300 (243–343	213 (157–290)	<0.001*	<0.001‡	<0.001‡
EBL (ml), median (i.q.r.)	75 (50–175)	75 (50–100)	100 (75–150)	0.014*	<0.001‡	0.042‡
Length of hospital stay (days), median (i.q.r.)	3 (2–4)	4 (3–6)	4 (4–6)	0.021‡	0.080‡	<0.001‡
VTE/pulmonary embolism	8 (7.5%)	8 (5.5%)	1 (0.7%)	0.547†	0.021†	0.005†
30-day readmission	30 (28.0%)	23 (15.9%)	10 (6.7%)	0.020†	0.015‡	<0.001‡
Follow-up from DLI closure (months), median (i.q.r.)	14.1 (4.4–27.5)	9.1 (3.7–23.3)	12.3 (5.9–28.6)	0.111*	0.033*	0.549*

Values are *n* (%) unless otherwise stated. IPAA, ileal pouch–anal anastomosis; BMI, body mass index; i.q.r., interquartile range; EBL, estimated blood loss; VTE, venous thromboembolism; DLI, diverting loop ileostomy. *Mann–Whitney *U* test; †Fisher's exact test; ‡χ^2^ test.

### Pouch failures

**Fig. 2:**
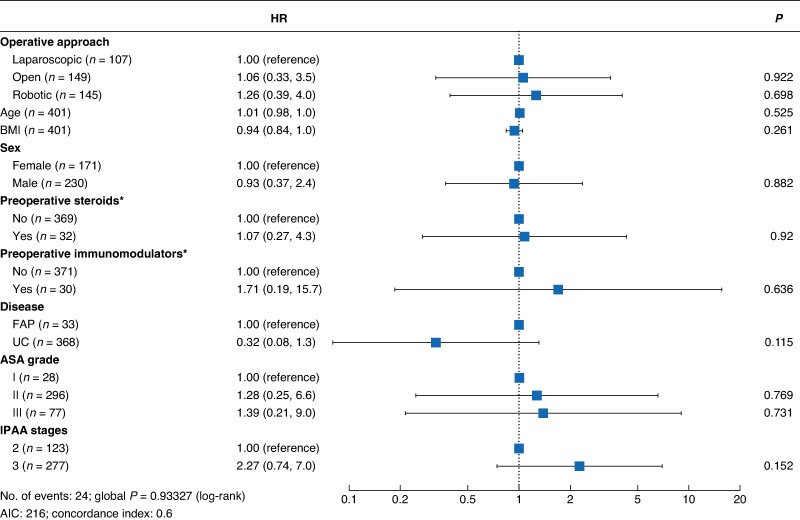
Forest plot showing Cox regression analysis of the effects of different variables on pouch failure *Preoperative use of steroids or immunomodulators within 30 days of surgery. BMI, body mass index; FAP, familial adenomatous polyposis; UC, ulcerative colitis; ASA, American Association of Anesthesiologists; IPAA, ileal pouch–anal anastomosis.

Time analysis of pouch failure (*Fig. 1*) revealed no significant difference in failure rates across the three surgical approaches. This observation held consistent throughout the follow-up period. Furthermore, Cox regression analysis confirmed these findings, demonstrating no correlation between pouch failure and factors such as surgical approach, preoperative or postoperative steroid use, preoperative immunomodulator use, underlying disease, or the number of stages involved in the procedure (*Fig. 2*).

### Regression analysis model

*Table 4* comprehensively summarizes the regression models constructed to analyse the primary outcomes of this study. Analysis of these primary outcomes revealed largely comparable results across most individual postoperative complications, including ileus, small bowel obstruction, superficial surgical site infection, pelvic abscess, and urinary tract infection for open, robotic, and laparoscopic IPAA approaches. However, notable differences emerged between the surgical techniques. Specifically, the open surgical approach had a lower incidence of VTE compared with both the robotic and laparoscopic approaches. In addition, open IPAA had fewer 30-day readmissions compared with both robotic and laparoscopic IPAA. All three surgical techniques resulted in similar rates of pouch failure and 30-day mortality. Regarding resource utilization, robotic IPAA was associated with a longer operative time than both open and laparoscopic approaches, whereas open IPAA was associated with a longer length of hospital stay than robotic IPAA. Conversely, robotic IPAA resulted in reduced EBL compared with open IPAA. Furthermore, when comparing surgical approaches, robotic surgery had a lower likelihood of conversion to open surgery than laparoscopic surgery.

**Table 4:** Summary of regression models for primary outcomes of open and robotic IPAA techniques relative to laparoscopy (*n* = 107) as the reference group

	Open IPAA technique		Robotic IPAA technique	
	Estimate	*P*	Estimate	*P*
Ileus	0.90 (0.828, 0.99)	0.047	0.911 (0.82, 1.00)	0.060
SBO	0.97 (0.92, 1.02)	0.323	1.00 (0.95, 1.06)	0.958
VTE	0.93 (0.89, 0.98)	0.01	0.98 (0.93, 1.03)	0.410
Superficial SSI	1.01 (0.98, 1.05)	0.514	1.00 (0.96, 1.04)	0.945
Pelvic abscess	0.98 (0.92, 1.04)	0.546	1.00 (0.94, 1.06)	0.988
UTI	0.98 (0.94, 1.02)	0.295	0.981 (0.94, 1.03)	0.435
30-day readmission	0.80 (0.73, 0.88)	<0.001	0.88 (0.81, 0.97)	0.008
30-day reoperation	0.95 (0.91, 1.00)	0.066	0.97 (0.93, 1.02)	0.179
30-day mortality	1.00 (0.99, 1.02)	0.598	1.00 (0.99, 1.01)	0.940
Pouch failure	1.02 (0.96, 1.09)	0.513	1.04 (0.98, 1.11)	0.210
30-day morbidity	0.92 (0.76, 0.96)	0.011	0.91 (0.80, 1.02)	0.107
Operative time	62.71 (36.08, 89.31)	<0.001	119.28 (93.06, 145.49)	<0.001
Length of hospital stay	1.64 (0.55, 2.59)	0.003	1.028 (0.02, 2.03)	0.045
Estimated blood loss	23.22 (−12.40, 58.80)	0.201	−52.41 (−88.66, −16.15)	0.005
Conversion to open	–	–	0.88 (0.83, 0.94)	<0.001

Values in parentheses are 95% confidence intervals. IPAA, ileal pouch–anal anastomosis; SBO, small bowel obstruction; VTE, venous thromboembolism; SSI, surgical site infection; UTI, urinary tract infection.

## Discussion

This study highlights the distinctions between surgical approaches for IPAA and demonstrates comparable pouch failure rates across all techniques. For patients treated at the tertiary referral centres included in this study, the pouch failure rate was 6.5% overall, with no statistically significant difference between robotic (8.4%), laparoscopic (4.7%), and open (6.1%) approaches. These findings are consistent with the lowest reported pouch failure rates in the literature^15,16^.

Achieving good outcomes could be attributed to several factors: a high volume of IPAA performed, the experience of the colorectal surgeons specializing in these procedures, a multidisciplinary approach to inflammatory bowel disease care involving gastroenterologists, radiologists, and pathologists, adherence to standardized perioperative protocols, and the use of enhanced recovery programs^2,17,18^.

Although robotic IPAA took longer than laparoscopic or open approaches, it had a significantly lower conversion rate to open surgery than laparoscopy (1.4 *versus* 17.8%; *P* < 0.0001). This is important because unplanned conversion is linked to worse outcomes, including longer hospital stays, increased complications like infections and bleeding, higher mortality rates, and increased costs^19–23^. This advantage in avoiding conversions may explain another key finding: robotic IPAA led to fewer 30-day readmissions than laparoscopy (15.9 *versus* 28.0%; *P* = 0.02). By minimizing the need for open conversion, robotic surgery could potentially reduce complications and readmissions, ultimately improving patient outcomes and mitigating costs of hospital stays, which may help justify its higher initial costs.

An interesting finding of this study was the higher rate of VTE and PE in the robotic and laparoscopic groups compared with the open group. This observation is contradictory to the established literature, which generally does not link robotic surgery to increased VTE/PE risk^24–26^. Guidelines from the American Society of Hematology suggest that robotic surgery may be associated with a lower risk of these complications^27^, and similar recommendations have been made by the American Society of Colon and Rectal Surgeons and the Society of American Gastrointestinal and Endoscopic Surgeons^28^. Despite this, the longer operative times associated with minimally invasive techniques are a well documented risk factor for VTE/PE^29,30^. Consequently, the observed difference may stem from prolonged operative time or other unmeasured factors, warranting further research to clarify this unexpected outcome.

The results indicate that the surgical approach for IPAA may not be a primary determinant of long-term pouch outcomes. This suggests that other factors are likely contributing to pouch failure^11^, underscoring a critical need for further research to better understand the complexities of pouch function and identify the specific causes of failure^31–34^. This conclusion is further supported by the observation that most pouch failures occurred more than 6 months after IPAA surgery, a period when the surgical approach itself is unlikely to be a major influence.

Several limitations inherent to this study's retrospective design warrant consideration. Potential selection bias and confounding exist, because surgeon discretion guided the choice of surgical approach. Although standardized protocols were in place, residual intersurgeon variability cannot be discounted. The interpretation of pouch failure rates requires caution due to the robotic group's shorter follow-up and the inclusion of learning-curve patients for this technique. This caution is amplified by the study potentially being underpowered for the primary outcome, increasing the risk of a type 2 error. Therefore, the observation that the robotic group had the highest (although non-significant) pouch failure rate must be viewed tentatively given the sample size. Furthermore, the study's reliance on data from only three centres within a single healthcare system may limit generalizability. Relatedly, data on 30-day readmissions were captured solely through this system's electronic health records. Although comprehensive for internal events across the sites, this approach introduces a potential for undercapture, because readmissions to outside institutions, particularly for patients who travelled significant distances for their initial surgery, would likely be missed.

Future research should focus on identifying factors beyond the surgical approach that contribute to pouch failure to further optimize patient outcomes after IPAA.

## Data Availability

Data are available from the corresponding author upon reasonable request.
